# Interest in Rhinoplasty and Awareness about its Postoperative Complications Among Female high School Students 

**Published:** 2012

**Authors:** Aliasghar Arabi Mianroodi, Mobina Eslami, Narges Khanjani

**Affiliations:** 1 *Shafa Hospital Clinical Research Unit and Assistant Professor, Dept of Otorhinolaryngology, Shafa Hospital, Kerman Medical University, Kerman, Iran*; 2 *Medical Student, Dept of Otorhinolaryngology, Shafa Hospital, Kerman Medical University, Kerman, Iran*; 3 *Assistant Professor, Department of Epidemiology and Biostatistics, Kerman University of Medical Sciences, Kerman, Iran*

**Keywords:** Awareness, Iran, Postoperative complications, Rhinoplasty

## Abstract

**Introduction:**

Rhinoplasty is a popular cosmetic surgical procedure. Informal statistics show that Iran has one of the highest rates of rhinoplasty in the world. However, rhinoplasty like any other surgery can have complications.

**Materials and Methods:**

In this cross-sectional study, 320 female students were selected by multistage cluster-stratified sampling from high schools in Kerman, Iran and each completed a questionnaire.

**Results:**

More than half of the students said they would like to undergo rhinoplasty. The main reasons for wanting rhinoplasty were beauty and because it is fashionable. However, more than half of the interested students did not know about the possible postoperative complications of rhinoplasty. There was no relation between interest in having rhinoplasty and parents’ education, city of birth or economic status.

**Conclusion:**

Many teenagers are interested in having rhinoplasty in Iran. As the number of teenagers and young adults who choose to have cosmetic surgery increases, surgeons should consider their expectations, motivations and awareness of postoperative complications before surgery.

## Introduction

Cosmetic plastic surgery has increased in the recent years, especially among young people. In 1999, in the U.S., board certified plastic surgeons performed about 600,000 plastic surgery procedures, of which approximately 25,000 were done on teenagers. Statistics show that in the U.S. the number of teenagers having cosmetic surgery doubled between 1994 and 1998 (1), while other statistics show that the number of cosmetic surgical and nonsurgical procedures performed between 1997 and 2003 almost tripled (2). One of the most popular plastic surgeries is rhinoplasty, in which the nose is reshaped in order to increase facial beauty. It is said informally that Iran has the highest rate of cosmetic rhinoplasty in the world (3). The increasing statistics relating to rhinoplasty in Iran can be attributed to several factors such as improvements in surgical techniques, the low cost and the desire of Iranian women to have pretty faces, as the rest of the body is covered due to Islamic regulations (3).

Although the incidence of rhinoplasty is increasing and it is generally regarded as a safe procedure, this surgery as with many others can have complications. A complication can be anything as simple as patient dissatisfaction, or as serious as death. These side-effects are not necessarily due to negligence by medical staff. Articles report that the rate of complications for this type of surgery varies from 4% to 18.8% (4). Skin and soft tissue complications have been reported to occur in up to 10% of cases. It is estimated that severe systemic or life-threatening complications occur in 1.7% to 5% of the surgeries (4). Complications of rhinoplasty are classified in four groups: intra-operative, and immediate, early and late postoperative complications (4). These complications are summarized in (Table 1) (4,5).

Considering the increasing rate of rhinoplasty surgeries especially in young women in Iran, it seems like this group are not fully aware of complications of the procedure and many have idealistic views about it. In this study we evaluated female teenagers’ views about rhinoplasty and its complications. 

## Materials and Methods

This was a cross-sectional study that included 320 female students selected from high schools in Kerman, Iran by multistage cluster stratified sampling. Schools were selected from two zones in this study. Zone 1 schools were located in the lower socioeconomic parts of the city and Zone 2 schools were located in the higher socioeconomic parts. In each zone, 5 schools were selected and 8 students were randomly selected from each grade, for a total of 32 students per school and 160 students from each zone. A questionnaire including demographic information, parents’ education, history of rhinoplasty in the family and perceptions about their nose shape and undergoing rhinoplasty was designed by the researchers. The face and content validity of the questionnaire was confirmed by expert opinion and its high reliability was confirmed by test-retest (r = 0.91) on 10% of the population. Each student completed a questionnaire. The data was entered and analyzed using MiniTab 15.

## Results

Study questionnaires were completed by 315 of the 320 female high school students, which is a response rate of over 98%. The mean ± standard deviation (SD) age of participants was 16.4 ± 1.1 and the minimum and maximum age was 14 and 19 years respectively. A total of 235 participants (75.3%) in this study were born in Kerman and the rest were born in other cities. Most of the students’ fathers had a diploma or higher level of education, while most of the mothers had a diploma. Among the participants of this study, 174 people (55.1%) knew at least one person who had had rhinoplasty among their close relatives or friends. The demographic information is summarized in (Table 2).

**Table 1:** The complications of Rhinoplasty

**Late postoperative complications**	**Early postoperative complications**	**Immediate postoperative complications**	**Intra-operative** ** complications**
Scar hypertrophy	Hemorrhage	Airway obstruction	Excessive bleeding
Polly Beak nasal deformity	Septal hematoma	Anaphylaxis	Tears of mucoperichondrial flaps
Synechiae Formation	Infection	Visual impairment	Buttonholing of skin
Septal perforation	Dehiscence of incisions		Cautery burns
Nasal valve Collapse	Persistent edema		Collapse of bony pyramid
Nasal stenosis	Skin necrosis		Disarticulation of upper lateral cartilage
Bossa Formation	Sequestra formation		Osteotomy complications
Recurrent Meningitis	Cardiovascular insufficiency		Perinasal trauma
Oleogranuloma	Cerebrospinal fluid rhinorrhea		
Dorsal Cyst	Contact dermatitis		
Aesthetic surgical misjudgments	Nasal blockage		
Persistent Psychological complications	Numbness and pain		
Dental complications	Olfactory Disturbances		
Gustatory rhinorrhea	Carotid-Cavernous fistula		
Human adjuvant disease	Reassurance Demand		
Lacrimal Fistula	Early psychological complications		
Enophthalmos and silent sinus syndrome			
Patient dissatisfaction			
Difficulty in Breathing			
Long-term impacts on the quality of life			
Sleep-related Breathing disorders			
Nasal crusting,Synechiae, and Discomfort			
Ozena or Advanced Atrophic Rhinitis Characterized by Chronic Crusting and Dysosmia Even resulting inAnosmia due to the Destruction of Olfactory cells.			

**Table 2:** The demographics of the female high school students participating in this study

Age (mean , SD)			16.4 , 1.05
City of Birth	Kerman		75.32 %
	Other cities		24.67 %
Father’s Education	Under high school diploma		14.63 %
	High school diploma		38.78 %
	Graduate diploma or bachelor		38.44 %
	Masters or Doctorate		8.16 %
Mother’s Education	Under high school diploma		20.20 %
	High school diploma		44.11 %
	Graduate diploma or bachelor		31.65 %
	Masters or Doctorate		4.04 %
Number of close relatives or friends whom have done a rhinoplasty		0	44.94 %
		1	24.8 %
		2	14.01 %
		3	8.92 %
		>4	7.33 %

Out of the student group 147 people (47.0%), which is just less than half of the sample population, were happy with the current shape of their nose, 118 people were not happy and 48 people said they do not care about the shape of their nose; 169 people (53.6%) said they would like to have rhinoplasty done. 

The reason for their tendency to want rhinoplasty was (in order of decreasing frequency): for beauty, to stay in fashion, to show off, pressure from family and friends and other reasons. The people who encouraged them to have rhinoplasty done were mainly the female family members including aunt, sister, mother, cousins and in fewer cases friend, father, brother and fiancé. Three students however mentioned "everyone". 

In total there were a 100 people (59.5%) who stated that they themselves were keen to have the surgery, and 68 people (40.5%) who had been persuaded by others. In this latter group, 33 people (32.39 %) had one person persuading them to undergo surgery and the rest had two or more people

persuading them.

The most important criterion for choosing a surgeon among the group was (in order of decreasing frequency): a surgeon whose surgical results they had seen before and who operates well, a person who charges less, a person who has lots of patients and is busy, a person with good manners, and other reasons. Some of the participants added reasons that were not mentioned in the questionnaire, such as being a family relative or being a trustworthy person. The attitudes of participants towards rhinoplasty are summarized in (Table 3).

Participants were also asked about rhinoplasty complications and if they thought that these complications may happen. The familiarity of the participants with the postoperative complications of rhinoplasty is summarized in (Table 4). 

The main reason for not being interested in undergoing rhinoplasty was (in order of decreasing frequency): the subjects liked their nose as it is, they think plastic surgery is in vain, a fear of surgery, a fear of complications, the high cost, and other reasons. Some of the participants mentioned reasons not listed in our questionnaire such as being ashamed to appear in public with a bandaged nose and a dislike for attracting attention. Some others mentioned that they believe whatever god has created is beautiful and should not be manipulated.

**Table 3:** Attitude toward rhinoplasty

How do you feel about your nose?	Happy	46.96 %
	Not Happy	37.70 %
	Don’t care	15.34 %
Do you want to do a rhinoplasty?	Yes	53.65 %
	No	46.35 %
Why do you want to do a rhinoplasty? For beauty		91.12 %
	To show off	4.73 %
	insist of friends or family	4.73 %
	To catch up with the mode	7.10 %
	Other reasons	10.56 %
What type of doctor would you like to operate you?	Doctor who charges less	17.75 %
	Doctor with good manners	11.24 %
	Doctor who has lots of patients and is busy	14.79 %
	Doctor who operates well	74.56 %
	Others	5.92 %
Why don’t you want to do a rhinoplasty?	Fear of operation	18.37 %
	Don’t have enough money	9.52 %
	Like my nose as it is	59.18 %
	Plastic surgery is no use	20.41 %
	Fear of side effects	15.65 %
	Other reasons	17.01 %

**Table 4:** Familiarity with some of the post operative complications of rhinoplasty

**Complication**	**Percent who did not know**
Skin discoloration	89.44 %
Breathing disorders	46.15 %
Recurrent nosebleed	81.66 %
Nose Blockage	75.15 %
Recurrent nasal mucosal irritation	82.74 %
Headache	87.5 %
Recurrent nausea and vomiting	89.48 %
Nasal discharge	91.07 %
Sensitivity to strong adores	89.29 %
Death	85.22 %
Need for reoperation	69.64 %
Dissatisfaction with new nose	66.86 %
Mismatch of new nose with the rest of the face	79.76 %

Among the people who did not want to undergo rhinoplasty, 38 people (26.2%) claimed that if their reason for not having rhinoplasty could be resolved, they would be interested in having the procedure done. 

The number of participants "interested in having rhinoplasty" increased with increases in the fathers' education, but the increase was not significant (P= 0.139). Participants whose mothers had between 2 and 4 years of university education were more keen to have rhinoplasty (63.4%) than others, but this increase was not significant either (P= 0.169). There was no significant association between city of birth (P = 0.83) or the socioeconomic level of the school (P=0.971) and tendency toward wanting rhinoplasty. There was no significant association between the socioeconomic level of the school and participants’ satisfaction with their nose shape (P= 0.296) either.

## Discussion

In this study just less than half of the participants were happy with the appearance of their nose and more than half of the participants knew someone among their relatives or close friends who had had rhinoplasty. In this population more than half of the people were keen to undergo rhinoplasty. This is in contrast to a study by Pearl et al. in California, where although 68% of the respondents knew someone who had undergone some kind of cosmetic surgery, only 30% would consider it for themselves (1). 

In our study the main reason for having rhinoplasty was beauty and to stay in fashion. In a Californian study of individuals who would choose cosmetic surgery, 90% said their motivation was to feel better about themselves (1). These results are almost in line with the results from Ghaleganji's study, which reported that among people interested in having rhinoplasties, obsessive and narcissistic characteristics were significantly higher than other personality types. This suggests that people with these personality types are eager to improve their self esteem through cosmetic surgery (6). Also, other studies suggest that psychological disturbances or poor general mental health can urge people toward rhinoplasty and therefore psychological assessment before the operation is recommended (7, 8). In our study among the people interested in having rhinoplasty, the person herself played the main role in deciding to have the operation and there were fewer participants who had been persuaded by others to have the surgery.

In this study more than half of the population did not know about the postoperative complications of rhinoplastic surgery. The complications mainly recognized by the participants were more trivial and included re-operation, dissatisfaction, and mismatch of the new nose with the rest of the face and their knowledge about the more serious complications such as headache, nausea and vomiting, and death was much less. Participants in the study who were keen to have rhinoplasty preferred to be operated by a surgeon who had performed previous successful operations, with other criteria such as low fees, having lots of patients, good manners and other factors being of less importance. In another study conducted by Harazi et al., the knowledge, ability and proficiency of the physician was of major importance to the patients, and the personal characteristics of the physicians, with the exception of knowledge and competency, were the least important factors (9).

Among the people who did not want to have rhinoplasty, most of them said the reason was that they are happy with their nose as it is and others mentioned reasons such as thinking plastic surgery is in vain, fear of the complications and the cost. In a study done in California, the main reasons why the participants would not choose cosmetic surgery for themselves were potential health risks, cost, and fear of a bad result (1), which is similar to the results of this study. However, more than a quarter of the non-willing participants of our study stated that if these obstacles were removed they would like to have rhinoplasty. Therefore, it can be predicted that in the near future with improvements in surgical techniques and an increase in the number of cosmetic surgeons and therefore decrease in the cost and complications of this surgery, the rate of cosmetic plastic surgery in Iran will increase.

Tendency toward desiring rhinoplasty in this study was not significantly related to parental education, although there did seem to be a slight increase as the level of the parents’ education increased. However there are reports indicating that the rate of cosmetic surgery or tendency to want surgery decreases as parental or family education increases (10, 11). We also did not observe any difference in the tendency to want rhinoplasty between people born in Kerman and people born in other cities. However, another study has reported that there was a significant difference between people born in the same city and other cities (12).

## Conclusion

As the number of teenagers and young adults who choose to have cosmetic surgery increases, understanding their attitudes, fears, and expectations seems essential for every surgeon (1). Moreover, understanding patients’ knowledge, motivations and expectations of the proposed plastic surgery is an important aspect of the clinical care of patients and their families (2). In addition, helping teenagers and young adults to increase their self-esteem, diagnosing personality problems beforehand and informing people of the possible complications of cosmetic surgery can help surgeons wisely choose their patients before operation.
